# Fundamental characteristics of voltage-modulated helium plasma for multi-element analysis in low-volume samples

**DOI:** 10.1007/s44211-026-00880-7

**Published:** 2026-02-17

**Authors:** Yuya Shimizu, Masaya Tahara, Yuwa Ando, Kai Fukuchi, Akane Yaida, Yukiko Moriiwa, Kazuhiro Morioka, Atsushi Shoji, Akitoshi Okino

**Affiliations:** 1 Laboratory for Future Interdisciplinary Research and Technology (FIRST), Institute of Science Tokyo, 4259-J2-32, Midori-ku, Nagatsuta, Yokohama, 226-8501 Japan; 2 School of Pharmacy, Tokyo University of Pharmacy and Life Sciences, Hachioji, 1432-1 Horinouchi, Hachioji, Tokyo 192-0392 Japan

**Keywords:** Optical emission spectroscopy, Mass spectrometry, Microplasma, Multi-element analysis, Voltage-modulated plasma, Low-volume samples

## Abstract

High-sensitivity multi-element analysis of low-volume samples remains a challenge in plasma-based analytical methods because optimal excitation conditions differ among elements and conventional steady-state plasma require relatively large sample volumes. In this study, we developed a voltage-modulated helium plasma in which the applied voltage was continuously varied over short time intervals to dynamically control the plasma energy state. The fundamental characteristics of plasma were evaluated in terms of gas temperature, emission spectrum, and excitation temperature. Under steady-state operation, plasma length, gas temperature, and excitation temperature increased with increasing applied voltage, confirming that the plasma energy state can be controlled by applied voltage. In the voltage-modulated plasma, emission intensity and excitation temperature responded rapidly to voltage changes, enabling the generation of plasma with different energy states within a short time. Notably, the excitation temperature, one of the key indicators of the plasma energy state, was dynamically varied over a wide range of approximately 1900–3600 K. In addition, voltage modulation effectively suppressed the overall increase in plasma gas temperature caused by Joule heating, while maintaining high-energy plasma states during high-voltage periods. These results demonstrate that voltage-modulated plasma generation enables rapid switching between energy states optimized for different elements, offering a practical strategy for high-sensitivity multi-element analysis with significantly reduced sample consumption. The proposed method is particularly suitable for the analysis of small-volume and heat-sensitive samples.

**Graphical abstract:**

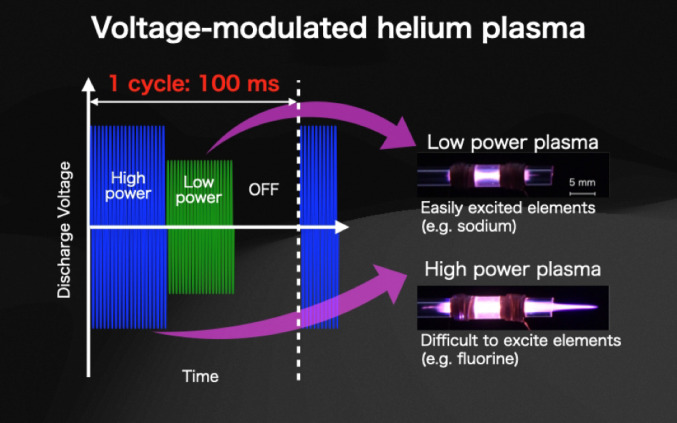

## Introduction

In recent years, highly sensitive analytical methods for multi-element analysis of low-volume samples have been increasingly required in fields such as medicine and environmental science. In the medical field, sensitive measurement of trace elements and metal ions in small biological samples—such as saliva [1, 2] and sweat [3, 4], which can be collected non-invasively—has been reported to be useful for early detection of health abnormalities, including diseases. In the environmental field, elemental analysis of low-volume samples, such as atmospheric particulate matter (PM2.5), has enabled the identification of emission sources, including industrial activities and soil-derived particles [5–7]. Therefore, high-sensitivity multi-element analytical methods for low-volume samples are particularly important in applications where the available sample amount is limited. The development of such analytical methods is expected to contribute to the advancement of diagnostic and environmental evaluation techniques.

Inductively coupled plasma optical emission spectroscopy (ICP-OES) [8, 9] and inductively coupled plasma mass spectrometry (ICP-MS) [10, 11] are widely used instruments for the highly sensitive analysis of various samples. These methods enable quantitative analysis of elements at concentrations ranging from parts-per-billion (ppb) to parts-per-trillion (ppt) [12], however, they generally require large sample volumes [13]. Therefore, reducing the sample volume consumption is essential for the analysis of biological samples such as sweat collected by natural perspiration or localized sampling. To address this issue, various approaches have been developed, including the sequential introduction of solution samples as microscale droplets [14, 15] and the use of microfluidic chips to minimize dead volume and introduce microdroplets into the plasma [16].

Furthermore, to enable elemental analysis of minute samples using plasma-based techniques, analytical methods employing microplasma generated within micrometer-scale channels have been actively developed [17–21]. These approaches achieve high analytical sensitivity for minute samples by reducing the plasma volume, thereby increasing both the energy density and the sample density within the plasma. However, in both conventional ICP-based methods and microplasma-based systems, modification of the plasma energy state to achieve high-sensitivity multi-element analysis [21–26] often prolongs the analysis time, which in turn leads to increased sample consumption. We therefore focused on the difference in excitation energies among elements. By temporally controlling the plasma energy state, it is possible to realize optimal excitation conditions for multiple elements within a short analysis time. This approach enables time-resolved utilization of different plasma energy states within a short measurement time and is thus well suited for multi-element analysis of minute samples.

In plasma-based analytical methods, elemental analysis is performed by exciting and ionizing atoms through energy transfer from the plasma. In plasma emission spectroscopy, each element has characteristic excitation and ionization energy levels. For example, the first ionization energies of sodium and fluorine are approximately 5.14 eV and 17.42 eV, respectively [27], demonstrating that the optimal plasma energy state differs significantly depending on the target element. Therefore, to achieve high-sensitivity elemental analysis, it is necessary to generate a plasma with an energy state appropriate for the excitation and ionization energies of each target element [19, 20]. Conventional plasma emission spectroscopy generally employs steady-state plasma operated under fixed generation conditions [22–24]. Maintaining a continuously high-energy plasma causes elements with low excitation energies to be readily over-excited, leading to self-absorption and non-linear emission behavior, which result in decreased analytical sensitivity. Conversely, operating the plasma at a lower energy state to optimize these elements leads to insufficient excitation efficiency for elements with higher excitation energies. Furthermore, continuous operation at high energy increases the electron density and discharge current, which can induce space-charge effects in the mass spectrometer and reduce the ion transmission efficiency of light mass elements. These analytical drawbacks indicate that a single steady-state plasma condition cannot simultaneously provide optimal analytical conditions for multiple elements. Therefore, temporal modulation of the plasma energy state is essential to achieve high analytical sensitivity for elements with different energy requirements within a short measurement time.

In this paper, we generated a helium plasma with rapidly varying energy states by continuously modulating the applied voltage over short time intervals. The gas temperature, emission characteristics, and excitation temperature of the resulting plasma were systematically evaluated.

## Experimental

### Measurement of voltage characteristics of plasma generated by steady-state voltage

**Fig. 1:**
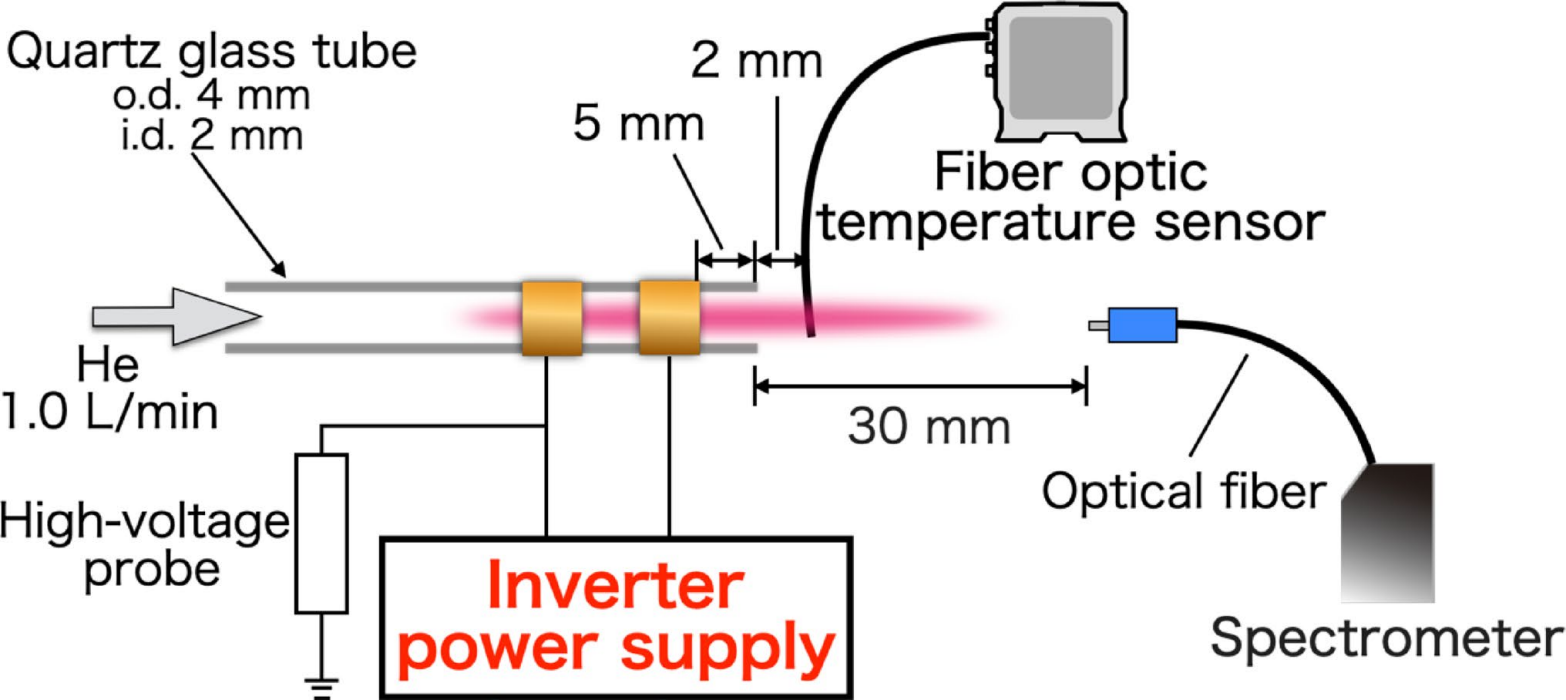
Experimental setup for helium plasma measurements

To compare the characteristics of plasma generated under steady-state applied voltages with those generated under time-modulated applied voltages, plasma generated at different applied voltages were first characterized. The experimental setup is shown in Fig. 1. The plasma source consisted of a quartz glass tube (i.d. 2 mm, o.d. 4 mm) equipped with two copper electrodes (width: 5 mm) positioned 5 mm from the tube tip and separated by a 5 mm gap. The electrodes were insulated with polyolefin heat-shrink tubing, Helium was introduced into the tube from the side opposite the electrode at a flow rate of 1.0 L/min. Plasma generation was achieved by applying a high voltage using an inverter power supply (PAE331C1-B-Z01, Ushio Inc, ) to the electrodes. In this study, helium was used as the plasma gas for highly sensitive multi-element analysis. Helium possesses the highest first ionization energy among all elements, and its metastable states have high energies of 19.82 eV and 20.62 eV, enabling efficient excitation and ionization of halogen elements that are difficult to analyze using argon plasma.

The applied voltage was varied under four conditions (4, 6, 8, and 10 kVpp) at a frequency of 50 kHz. The minimum applied voltage was determined to be 3.4 kVpp, which corresponds to the lowest voltage at which the fabricated plasma source was capable of generating a helium plasma. The applied voltage was monitored using an oscilloscope (DPO4104, Tektronix) and a high-voltage probe (HVP-30pro, Pintex). The plasma energy state was evaluated by measuring optical emission spectra. Emission from the plasma was collected using an optical fiber and introduced into a multichannel spectrometer (Maya 2000 Pro, Ocean Optics). The fiber tip was positioned 30 mm coaxially from the plasma source tip to prevent direct discharge into the fiber. The plasma gas temperature was measured using a fiber optic temperature probe (PRB-G40-02 M-ST-C, Osensa Innovations) connected to a fiber optic temperature transmitter (FTX-200-LUX+, Osensa Innovations). The probe was positioned 2 mm from the plasma source tip to minimize disturbance of the discharge.

### Characterization of voltage-modulated plasma

A plasma with a stepwise-modulated voltage was generated, and its gas temperature and emission characteristics were investigated. The applied voltage was varied in three steps within a single 100 ms cycle. The appllied voltage was modulated between 10, 4, and 0 kVpp for durations of 40, 30, and 30 ms, respectively. The power supply used in this study is capable of three-stage modulation at frequencies of 1–10 Hz. Therefore, three-stage voltage modulation was performed at 10 Hz (100 ms), which is the fastest modulation time available with this power supply. The duration of each voltage step was set to 40, 30, and 30 ms to clearly distinguish the transition points between each voltage.

In addition, the applied voltage modulation sequence (10–4–0 kVpp) was selected based on the consideration that plasma generation at lower voltages may not be reliable during the initiation phase. Accordingly, the sequence was designed to begin at 10 kVpp, where stable plasma generation can be consistently achieved, thereby avoiding the risk of unsuccessful plasma generation at 4 kVpp during repeated modulation cycles. Furthermore, assuming that stable plasma generation can be maintained at 4 kVpp, even when the sequence is reversed, no significant differences in plasma characteristics or analytical performance are expected between the two modulation orders.

The experimental setup was identical to that shown in Fig. 1, except for the spectroscopic system. For steady-state plasma generated at a constant voltage, a multichannel spectrometer was used for emission measurements. However, when the applied voltage was rapidly modulated, data acquisition with a multichannel spectrometer became time-consuming, making time-resolved spectroscopic measurements difficult. Therefore, a Czerny–Turner-type sequential spectroscope (grating: 1800 grooves mm⁻¹) was employed. Emission signals were detected using a photomultiplier tube (R3896, Hamamatsu Photonics). To maximize the detection efficiency of the photomultiplier tube, which exhibits peak sensitivity near 450 nm, five helium emission lines were selected for analysis: He I 402.62 nm, He I 447.15 nm, He I 471.32 nm, He I 492.19 nm, and He I 502.57 nm.

## Results and discussion

### Gas temperature of plasma generated by a steady-state voltage

**Fig. 2:**
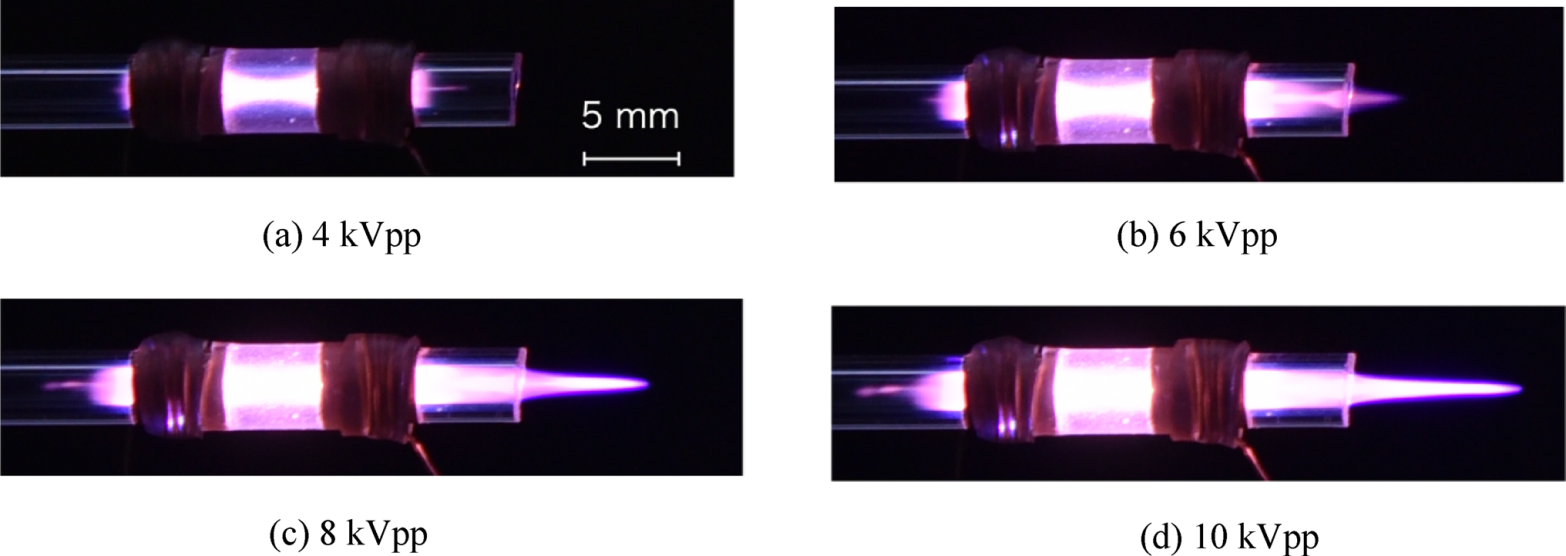
Helium plasma generated at each applied voltage

Figure 2 shows the appearance of helium plasma generated at different applied voltages. As the applied voltage increased, the plasma length extended further from the plasma source. This behavior is consistent with previous reports indicating that, in plasma generated under an axial electric field, the plasma length increases with increasing applied voltage [28, 29]. This extension can be attributed to the enhanced axial electric field strength at higher voltages, which increases the energy transferred to atoms and charged species within the plasma. As a result, ions and excited species can retain sufficient energy to propagate toward the outer regions of the plasma. The elongation of the plasma results in an increased residence time of analyte species within the plasma, thereby enhancing the number of collisions with energetic plasma species [30]. Consequently, excitation and ionization efficiencies are enhanced, leading to improved analytical sensitivity. However, during radial observation, the plasma length varies with the discharge voltage, causing the optimal position for emission measurement to shift. Therefore, for high-sensitivity analysis using the plasma source developed in this study, observation in the coaxial direction is considered necessary.

**Fig. 3:**
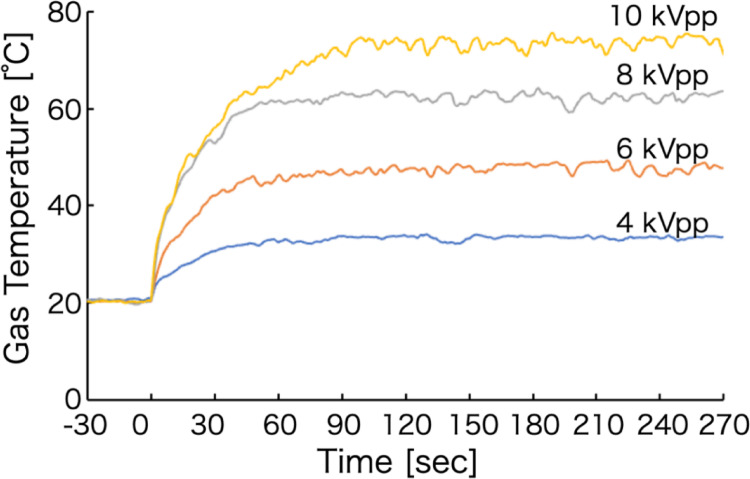
Time evolution of gas temperature in helium plasma generated at each applied voltage

Figure 3 shows the temporal evolution of the plasma gas temperature measured at each applied voltage. The average gas temperature was calculated from data acquired over a 120 s interval between 150 and 270 s after plasma generation, when the temperature had reached a steady state. For all applied voltages, the gas temperature required approximately 60–90 s to stabilize after plasma generation. This behavior is attributed to combined heating effects arising from energy input from the power supply and Joule heating of the plasma source components, including the electrodes. The average gas temperature of the helium plasma increased from approximately 33.5 °C at 4 kVpp to approximately 73.7 °C at 10 kVpp. This increase can be explained by the higher electric field strength at elevated voltages, which imparts greater energy to gas-phase particles. In addition, the accompanying increase in discharge current enhances Joule heating, further contributing to the observed temperature rise [31]. Thermal stability was evaluated using the %RSD of the gas temperature during the steady-state interval (150–270 s). A %RSD of 0.76–1.78% was obtained, indicating stable plasma generation.

### Spectral characteristics of plasma generated by a steady-state voltage

**Fig. 4:**
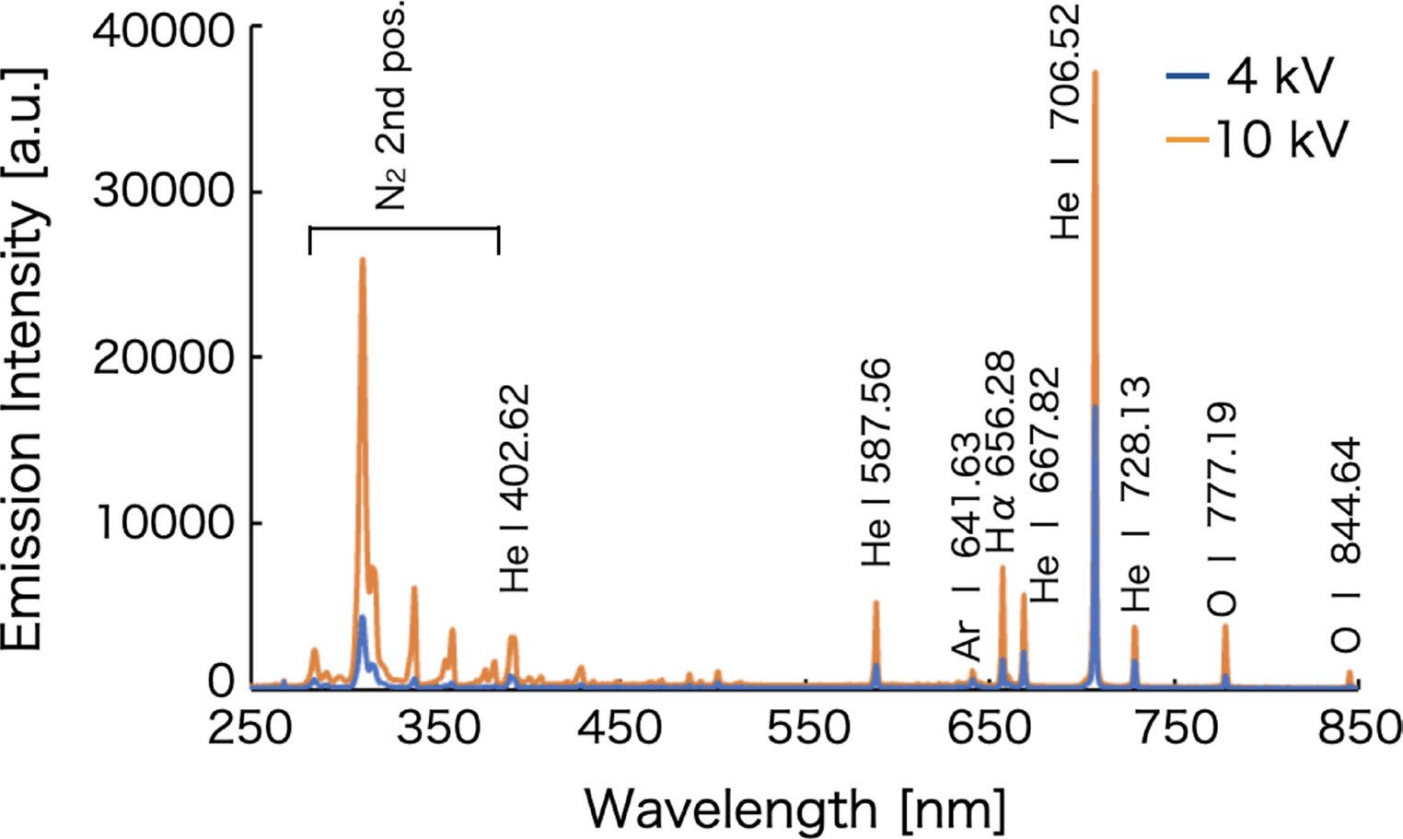
Emission spectra of helium plasma generated at each applied voltage

Figure 4 shows the emission spectra of helium plasma generated at applied voltages of 4 and 10 kVpp. For both conditions, helium atomic emission lines were observed at 402.62, 587.56, 641.63, 667.82, 706.52, and 728.13 nm. In addition, the hydrogen atomic emission line (Hα) was detected at 656.28 nm. The emission intensities of all observed helium atomic lines increased with increasing applied voltage. The He I emission at 706.52 nm exhibited the highest intensity, showing an increase by a factor of approximately 2.2 when the applied voltage was raised from 4 to 10 kVpp. This enhancement can be attributed to the increased energy input to the plasma and the resulting increase in plasma density at higher applied voltages. Moreover, as shown in Fig. 2, the plasma length increased with increasing appllied voltage. Because the emission spectra were collected along the axial direction of the plasma, this elongation likely contributed to the observed increase in emission intensity.

In contrast, the emission intensity of the N₂ second positive system, observed at several wavelengths in the range of 300–400 nm, increased more markedly than that of the helium atomic lines. This behavior is attributed to the fact that, at an applied voltage of 4 kVpp, the plasma remained confined within the quartz glass tube. However, at higher voltages, the plasma extended beyond the tube and interacted with the ambient air. As a consequence, nitrogen molecules in the surrounding atmosphere were efficiently excited, resulting in a pronounced increase in the N₂ emission intensity.

### Excitation temperature of plasma generated by a steady-state voltage

**Fig. 5:**
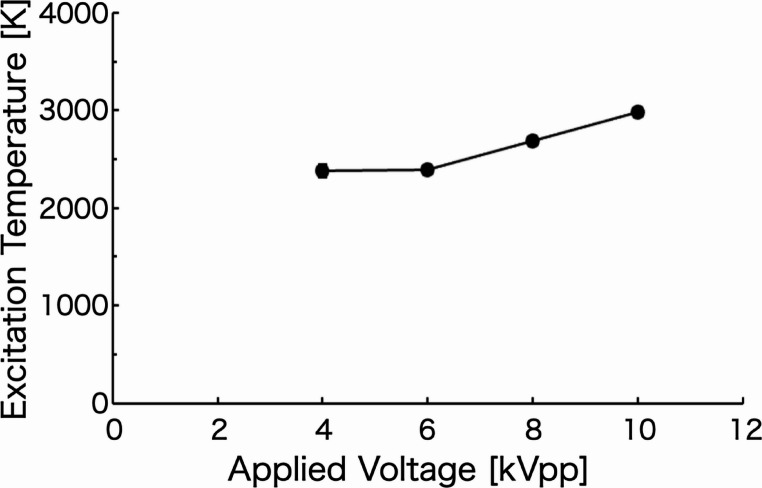
Excitation temperature of helium plasma generated at each applied voltage

Figure 5 shows the dependence of the excitation temperature on the applied voltage, as determined from the relative intensity ratios of the measured helium atomic emission lines. The excitation temperature of the helium plasma increased from approximately 2400 K to 3000 K with increasing applied voltage. The observed increase in excitation temperature is attributed to the enhanced energy transfer to gas-phase particles resulting from the higher appllied voltage.

These results demonstrate that the excitation temperature of the helium plasma can be systematically controlled by adjusting the voltage, thereby enabling the generation of plasma with different energy states.

### Gas temperature of voltage-modulated plasma with rapidly varying applied voltage

**Fig. 6:**
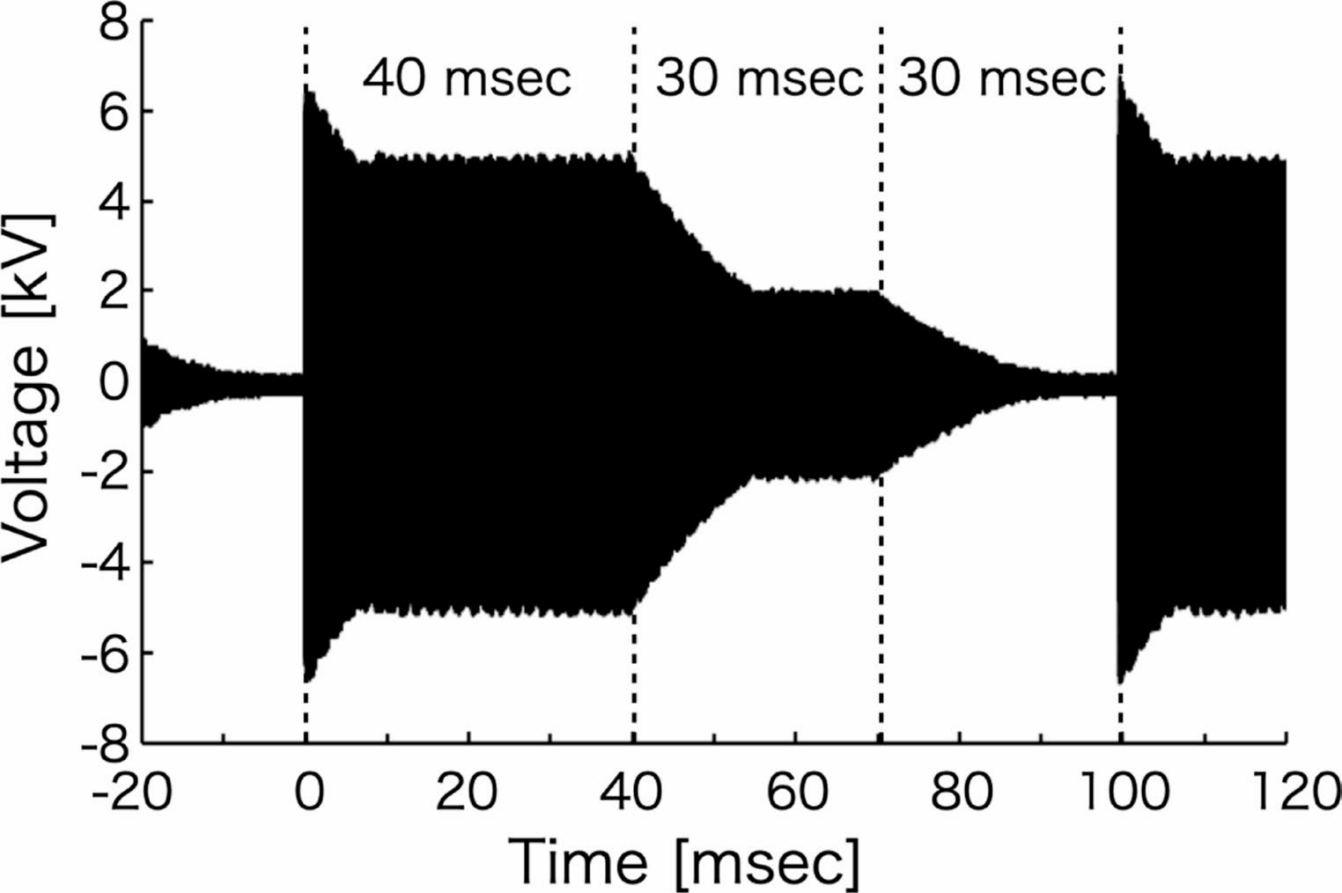
Applied voltage of voltage-modulated helium plasma

Figure 6 shows the voltage waveform applied for the generation of the voltage-modulated helium plasma. Although the applied voltage was supplied at the preset value, a transient overshoot exceeding the set voltage was observed at the onset of voltage application. The voltage rise time was evaluated based on the rise time definition from 10 to 90% of the set value [32]. The measured rise time was approximately 0.48 ms.

**Fig. 7:**
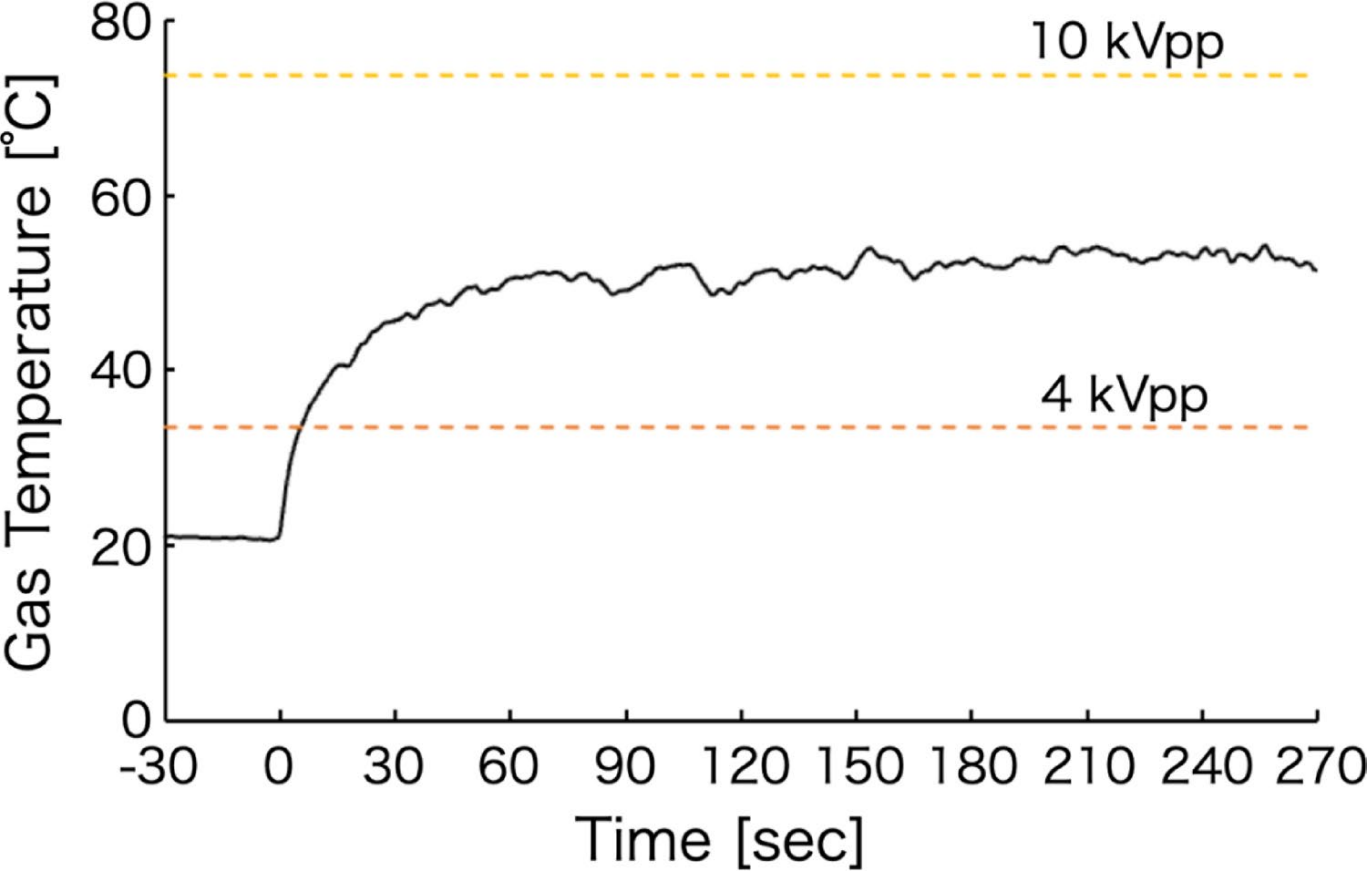
Time evolution of gas temperature in voltage-modulated helium plasma

Figure 7 shows the temporal evolution of the gas temperature for the voltage-modulated helium plasma. For comparison, Fig. 7 also includes the average gas temperatures measured at each constant applied voltage, as discussed in the previous section. The thermometer used in this study has a time resolution of 250 ms, which is insufficient to resolve the rapid, three-step temperature changes of the plasma gas, whose response time is in the order of several to tens of milliseconds.

It was confirmed that, as in the case of steady-state plasma generation, approximately 60 s were required for the gas temperature to reach a steady state after plasma generation. The average gas temperature was therefore calculated from data acquired over a 120 s interval between 150 and 270 s after stabilization. The resulting average gas temperature of the voltage-modulated helium plasma was approximately 52.8 °C. This value closely agrees with 53.6 °C, which corresponds to average gas temperatures measured at constant applied voltages of 4 kVpp (33.5 °C) and 10 kVpp (73.7 °C). These results indicate that modulation of the voltage enables the generation of a high-energy plasma while suppressing the overall increase in plasma gas temperature. This behavior arises because energy input to the plasma and Joule heating due to discharge current are enhanced during high-voltage periods and reduced during low-voltage or zero-voltage periods as a result of temporal voltage modulation. Moreover, Joule heating in plasma sources has been reported to cause degradation of electrodes and plasma source components [33]. In the present voltage-modulated plasma, the stepwise variation of the applied voltage reduces the cumulative Joule heating during high-voltage operation, thereby mitigating thermal stress on the electrodes and plasma source. Consequently, this approach has the potential to extend plasma source lifetime while maintaining or improving analytical sensitivity. Thermal stability was evaluated using the %RSD of the gas temperature during the steady-state period (150–270 s). The %RSD was 1.45%, indicating stable operation. Within this steady-state interval, differences in the magnitude of temperature fluctuations were observed as the plasma generation time progressed. Therefore, the %RSD values were compared for two 60 s intervals: 150–210 s and 210–270 s. The results were approximately 1.56% for 150–210 s and 1.10% for 210–270 s. These findings indicate that, over time, heating of the plasma source due to the discharge and heat exchange with the surrounding air gradually approached thermal equilibrium, resulting in improved analytical stability.

### Spectral characteristics of voltage-modulated plasma

**Fig. 8:**
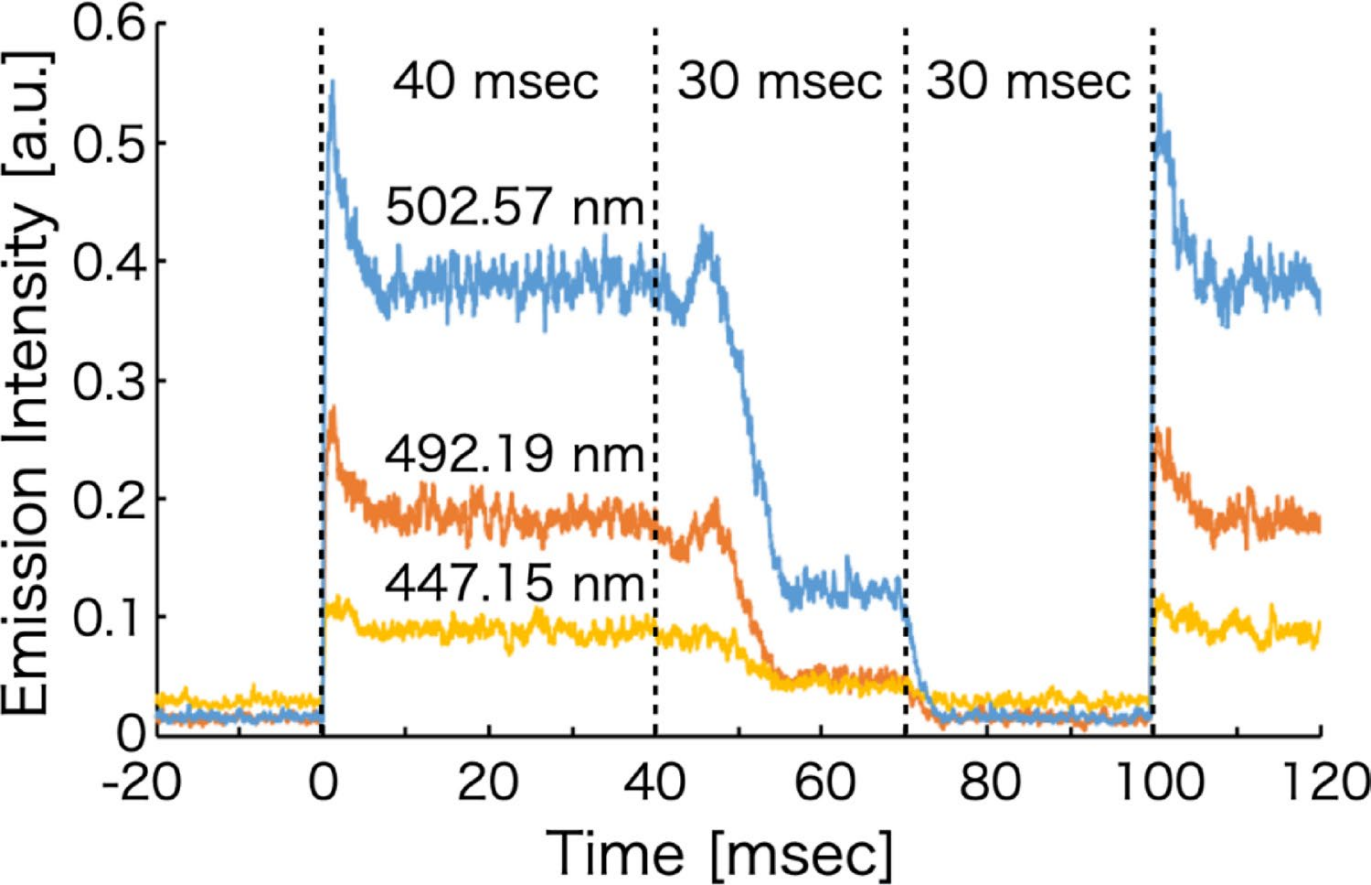
Time evolution of the emission intensity of voltage-modulated helium plasma

Figure 8 shows the temporal evolution of the emission intensities of the voltage-modulated helium plasma. The emission intensity at each measured wavelength varied synchronously with changes in the voltage. When the rise time was evaluated using the same 10–90% definition as that applied to the voltage waveform, wavelength-dependent delays were observed, ranging from approximately 0.81 to 1.45 ms. In addition, during the rising phase of the voltage, the emission intensity increased at different rates depending on the wavelength, particularly in regions where the voltage was elevated. For the He I lines at 447.15 and 502.57 nm, the emission intensity exhibited a transient increase even as the voltage decreased from 10 kVpp. This behavior is attributed to emission arising from recombination processes occurring during the voltage decay. Such recombination may generate pulse-like emission signals during analysis and could potentially affect the measurements. Therefore, for stable analytical operation, it is suggested that each modulated voltage step should be maintained for a sufficiently long duration rather than applying abrupt, pulse-like modulation. In contrast, although the voltage decreased gradually when transitioning from 4 kVpp to 0 kVpp, the emission intensity dropped rapidly. This rapid decrease is explained by the fact that the minimum voltage required to sustain helium plasma in the plasma source was approximately 3.4 kVpp. Below this threshold voltage, stable plasma generation could not be maintained, resulting in an abrupt loss of emission.

### Excitation temperature of voltage-modulated plasma

**Fig. 9:**
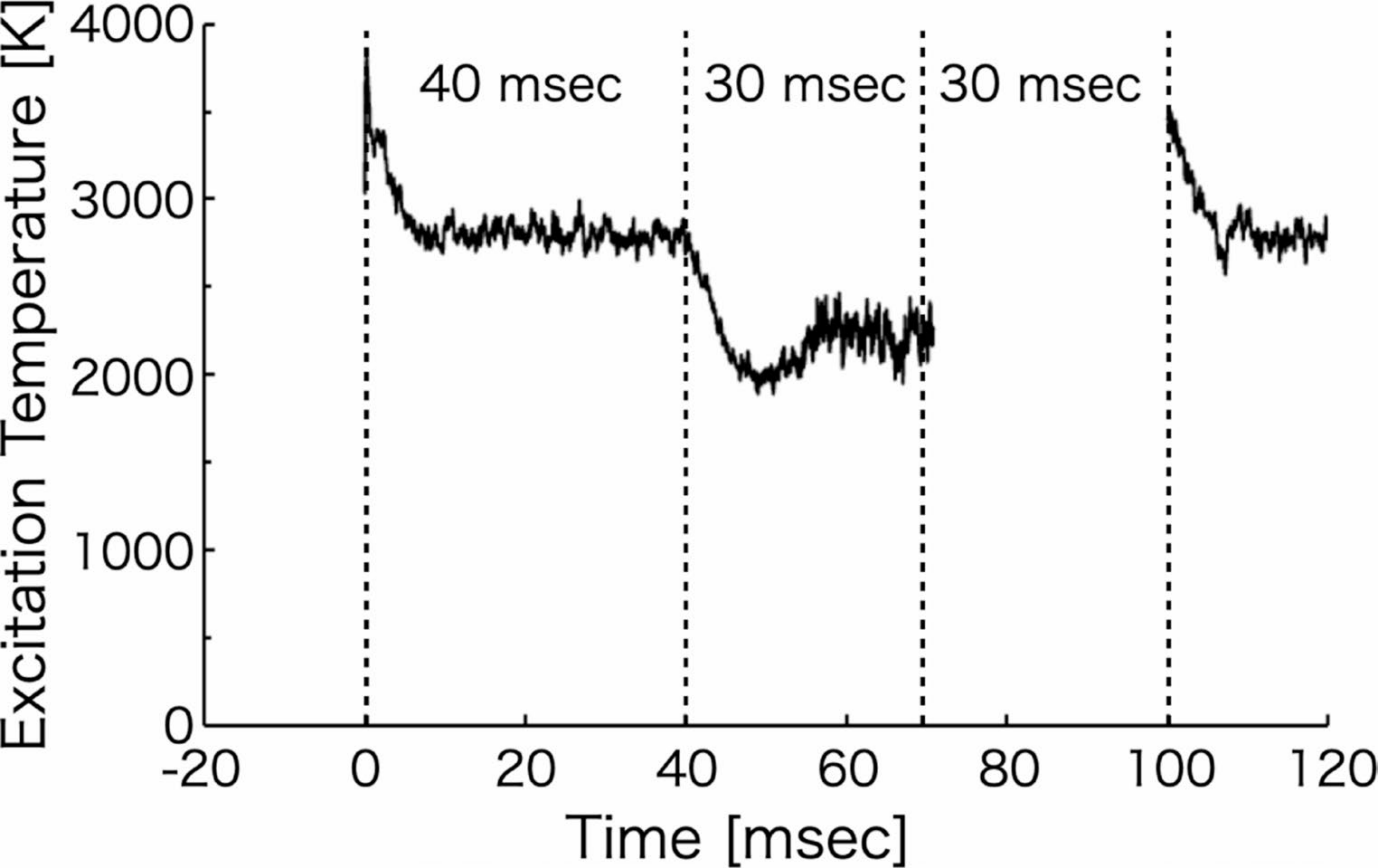
Time evolution of excitation temperature in voltage-modulated helium plasma

Figure 9 shows the temporal evolution of the excitation temperature calculated from the emission intensities measured at each wavelength. Because the excitation temperature was derived from intensity ratios of multiple emission lines, data points corresponding to zero emission intensity were excluded from Fig. 9. The excitation temperature of the voltage-modulated helium plasma varied between approximately 1900 and 3600 K in response to changes in the applied voltage. The larger variation in excitation temperature compared with that of the steady-state plasma is attributed to the effects of voltage overshoot and recombination processes.

When the time delay was evaluated using the same definition as that applied to the voltage and emission intensity, the rise time of the excitation temperature was found to be slower than that of the emission intensity, with a value of approximately 1.69 ms. These results clearly indicate that the rise times follow the order: applied voltage < emission intensity < excitation temperature. This sequence is consistent with the plasma generation process [34], in which electrical breakdown occurs once the applied voltage exceeds the breakdown voltage, followed by plasma formation and subsequent excitation of gas-phase atoms.

The decay time of the excitation temperature was approximately 3.27 ms. Furthermore, assuming a discharge duration of 10 ms under steady-state voltage operation and taking into account the measured rise and decay times, the maximum repetition frequency for realizing three-stage plasma characteristics was estimated to be approximately 24 Hz. These findings demonstrate that continuous modulation of the voltage enables dynamic control of the plasma energy state, indicating strong potential for high-sensitivity multi-element analysis of minute samples.

## Conclusion

In this study, a voltage-modulated plasma was generated to enable high-sensitivity multi-element analysis of low-volume samples by continuously varying the applied voltage over short time intervals. The gas temperature, emission characteristics, and excitation temperature of the generated plasma were systematically evaluated for both steady-state plasma and voltage-modulated plasma. Under steady-state voltage operation, the plasma length, gas temperature, and excitation temperature increased with increasing appllied voltage, demonstrating that the plasma energy state can be controlled by the applied voltage.

In the voltage-modulated plasma, in which the voltage was varied stepwise within a short time period, the emission intensity and excitation temperature responded rapidly to changes in the applied voltage. As a result, plasma with different energy states could be generated within a short time. In addition, voltage modulation was shown to suppress the overall increase in plasma gas temperature caused by Joule heating, while maintaining the high-energy plasma state during high-voltage operation. This characteristic is advantageous for mitigating thermal degradation of electrodes and plasma source components, thereby contributing to stable long-term operation.

These results demonstrate that continuous modulation of the voltage enables rapid switching between plasma energy states optimized for different elements. This approach has strong potential for highly sensitive multi-element analysis with a significantly reduced sample consumption. When combined with a microdroplet injection system [14, 15], a single droplet (14 pL) can be analyzed under three different plasma conditions. However, the plasma must be modulated precisely at the moment the droplet is introduced into the plasma. Therefore, the introduction system must be designed to ensure a constant and reproducible flight time from droplet ejection to entry into the plasma. We are currently developing an introduction system in which the time from droplet ejection to plasma introduction is kept constant, along with a synchronization system that coordinates droplet introduction with plasma modulation. In addition, when using a nebulizer operated at a flow rate of 1 mL min⁻¹, as commonly employed in conventional ICP-OES, analysis under three different plasma conditions is expected to be feasible with a sample volume of approximately 1.67 µL. Potential applications include biological analysis of sweat collected by natural perspiration or localized sampling, environmental analysis of adhered particulate matter, and materials analysis of thin films. Furthermore, the suppression of plasma gas temperature suggests that this method may also be applicable to the non- destructive analysis of heat-sensitive samples, such as biological materials.

## Data Availability

The datasets generated and analyzed during the current study are available from the corresponding author upon reasonable request.
